# Translation quality control in *Pseudomonas aeruginosa*: current knowledge and perspectives

**DOI:** 10.1093/femsre/fuag018

**Published:** 2026-04-15

**Authors:** Bastien L’Hermitte, Reynald Gillet, Christine Baysse

**Affiliations:** University of Rennes, CNRS, Institut de Génétique et Développement de Rennes (IGDR) UMR6290, 35000 Rennes, France; University of Rennes, CNRS, Institut de Génétique et Développement de Rennes (IGDR) UMR6290, 35000 Rennes, France; University of Rennes, CNRS, Institut de Génétique et Développement de Rennes (IGDR) UMR6290, 35000 Rennes, France

**Keywords:** *Pseudomonas aeruginosa*, ribosome quality control, antibiotic resistance, ribosome protection, ribosome rescue, ribosome hibernation

## Abstract

The fitness and virulence of *Pseudomonas aeruginosa* rely on its ability to maintain a functional pool of ribosomes, which are essential for protein synthesis. This review explores the intricate ways of ribosome protection, rescue, and hibernation, by which *P. aeruginosa* preserves ribosome functionality under stress. These processes enhance the adaptability and resistance of this pathogen to ribosome-targeting antibiotics and present significant challenges to current therapeutic strategies. By highlighting recent discoveries and identifying promising directions for future research, this review aims to explore potential targets for innovative drug discovery.

## Teaser

This review addresses the various mechanisms for maintaining ribosome functionality under diverse conditions and opens new research perspectives on the adaptation of *Pseudomonas aeruginosa* to stress and antibiotics.

## Introduction

Canonical translation is a fundamental cellular process wherein mRNA is decoded by the ribosome to synthesize proteins. To ensure optimal performance, it is crucial to maintain a readily available pool of functional ribosomes. Various stress conditions, such as the presence of ribosome-targeting antibiotics, physicochemical stresses, or starvation, can temporarily lead to a reduction in this pool or pressure bacteria to adapt by developing mechanisms to either restore ribosome availability or store them for later use (Njenga et al. 2023). Under many different growth conditions, ribosome concentrations limit growth rates (Scott et al. 2010), making the maintenance of the pool of functional ribosomes crucial, especially when considering the time and high energy requirements of ribosome biogenesis.

**Figure 1:**
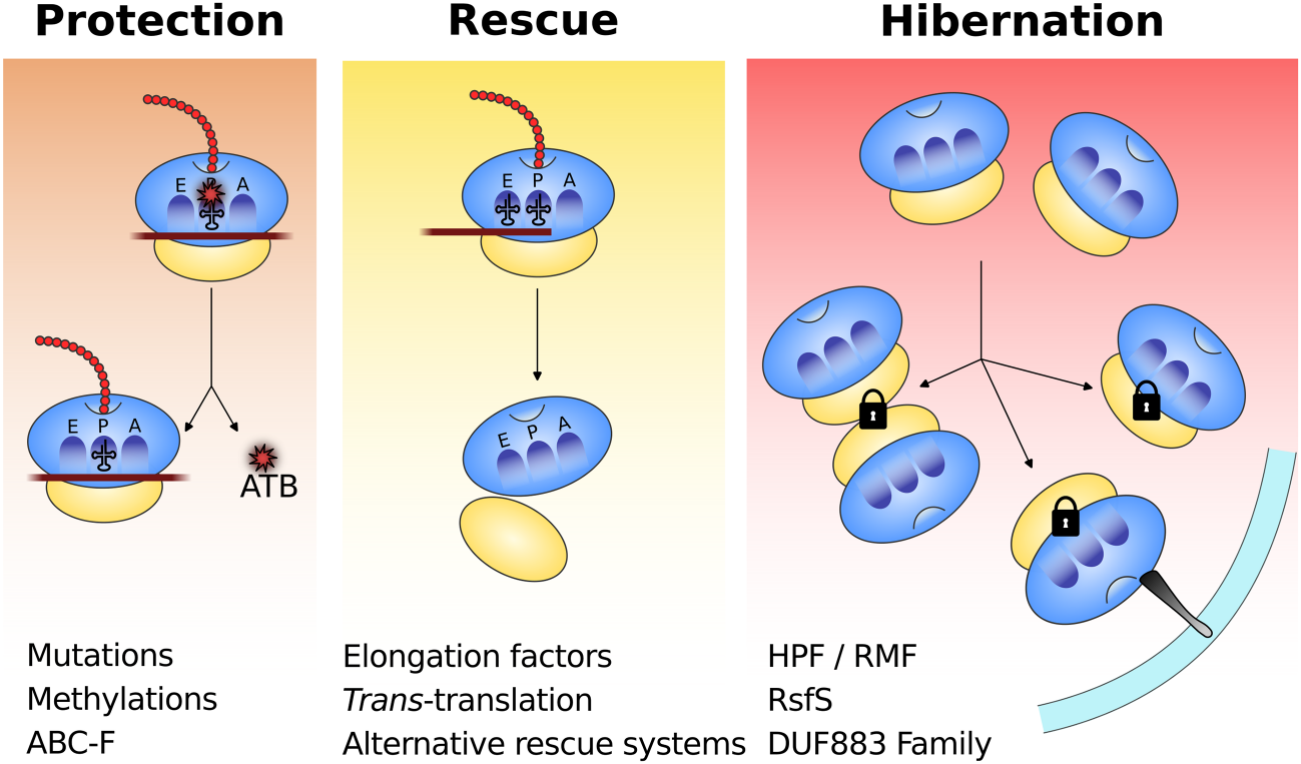
Schematic representation of the mechanisms involved in maintaining a functional ribosome pool. Based on gene similarities with mechanisms characterized in other bacterial species, and with studies specifically conducted in *P. aeruginosa*, the three stages of translational maintenance (protection, rescue, and hibernation) have been identified in this organism. The main factors involved in each process are listed on the figure. See text for details. E: Exit site; P: Peptidyl-tRNA binding site; A: Aminoacyl-tRNA binding site; ATB: antibiotic.

For existing ribosomes, mechanisms to maintain an optimal translation rate can be divided into three key components: protection, rescue, and hibernation (Fig. 1).

This review focuses on these three translation quality control strategies in *Pseudomonas aeruginosa*. Each process not only mitigates potential threats from environmental stresses and antibiotics but also contributes to the regulation of canonical translation quality. In all cases, the goal is to preserve a canonically functional ribosome capable of efficiently translating mRNAs into proteins. Ribosome protection involves (i) stable changes such as mutations in ribosomal components or methylation of rRNA that reduce antibiotic binding, and (ii) protein-mediated protection relying on specialized factors that transiently associate with the ribosome to shield antibiotic binding sites without permanently modifying its structure (s*Connell et al. 2003*, for a review). Ribosome rescue, including *trans*-translation and alternative rescue factors, recover ribosomes trapped on problematic mRNAs. Finally, ribosome hibernation sequesters translationally inactive but intact ribosomes, preserving them for future use. While these mechanisms are not exclusively linked to antibiotic exposure, many of them contribute directly or indirectly to bacterial survival under ribosome-targeting antibiotics and are therefore of particular interest as potential drug targets for emerging multidrug-resistant (MDR) pathogens.

Among pathogens, *P. aeruginosa*, a widespread γ-proteobacterium, poses a significant concern due to its capacity to opportunistically infect humans and its resistance to commonly used clinical antibiotics, including carbenicillin. The emergence of carbenicillin-resistant *P. aeruginosa* strains (CRPA) emphasizes the pressing need for innovative antibiotics to address this growing threat (Tacconelli et al. 2018). This pathogen is still among the high-priority pathogens that the WHO identified as 12 bacterial species and families in urgent need of new antibiotics (Miller and Arias 2024). However, the high mortality rate among immunocompromised individuals and within healthcare settings underscores the ongoing need for innovative approaches to mitigate the impact of CRPA on health care. One of the most problematic aspects of *P. aeruginosa* infections is their ability to persist in the host, thereby creating a reservoir for future infections (Rosa et al. 2025). One particularly vulnerable population is patients with cystic fibrosis (CF). *P. aeruginosa* infects these patients by exploiting the accumulation of mucus and the damage to lung tissues caused by the deficiency in the cystic fibrosis transmembrane conductance regulator (CFTR) channel. Today, a highly effective CFTR modulator therapy, elexacaftor/tezacaftor/ivacaftor, significantly improves the respiratory symptoms. Research indicated a reduction in lung bacterial burden post-treatment (Gushue et al. 2023, Sheikh et al. 2023, Ledger et al. 2024), but with less impact on chronic *P. aeruginosa* populations (Hisert et al. 2017, Sosinski et al. 2022, Nichols et al. 2023). Treated patients continued to be infected with the same strain of *P. aeruginosa* in both their upper and lower respiratory tracts, with the emergence of new variants. This highlights the ongoing importance of treating CF airways against *P. aeruginosa*, even with highly effective CFTR modulator therapy (Armbruster et al. 2024).

This pathogen is also capable of persisting in nutrient-poor environments, such as water intended for human consumption. Contamination of drinking water networks by this pathogen was reported by a French study from eight geographically distant cities. Five out of the eight networks tested were found to be contaminated with *P. aeruginosa* when tested using a culture-based method that resuscitates persister cells. Most isolates possessed a genomic island conferring copper-ion tolerance, facilitating their spread in such environments (Horikian et al. 2024).

Therefore, combating *P. aeruginosa* infections has become a formidable challenge, prompting the need for novel strategies. A better understanding of the remarkable adaptive abilities of this pathogen, as well as the role of translation maintenance systems in this capacity, is appealing. We aim to provide a state-of-the-art overview of the knowledge concerning the quality control systems of protein synthesis (translation) in *P. aeruginosa* with an emphasis on their possible roles in fitness and virulence.

## Preserving ribosome function: target alteration and protection proteins

Numerous antibiotics used in clinical settings are designed to disrupt bacterial ribosomes and hinder protein synthesis, especially during the elongation phase. The most used ribosome-targeting antibiotics against *P. aeruginosa* are aminoglycosides, tetracyclines, macrolides, and chloramphenicol. However, *P. aeruginosa* has evolved multiple resistance mechanisms that preserve ribosome function, mitigating the efficacy of these drugs. These include mutations that alter ribosomal targets, enzymatic modifications such as rRNA methylation, and the action of ribosome protection proteins (RPP). This section examines these molecular mechanisms, focusing on their role in antibiotic resistance and their broader relevance in defending the ribosome against stress.

### Ribosome mutations

Mutations can arise and be selected during antibiotic exposure, leading to the adaptation of a less sensitive population. These mutations can therefore be considered as conferring ribosomal protection relative to the ribosomal state of the original population. Aminoglycoside resistance resulting from ribosomal protein mutations in *P. aeruginosa* clinical isolates from early-stage lung infections in CF patients has been reported (Marvig et al. 2015). Cryo-EM structural analysis of ribosomes from both wild-type and mutant strains revealed changes in the conformation of rRNA helix H69 and protein uL6 (*rplF*, PA4248), both of which interact with the initiation factor IF2. These mutations do not involve a direct modification of the antibiotic-binding site but instead occur at a site 5 nm away (Halfon et al. 2019). This highlights the importance of considering ribosome mutations in the context of the whole ribosome rather than only the binding site of the antibiotic, and possibly through alterations in interactions with translation factors.

Multiple clinical studies have shown that CF patients chronically infected with *P. aeruginosa* benefit from azithromycin (AZM) treatment. This benefit has been largely attributed to the anti-inflammatory effects of AZM, which mitigate tissue damage (Zimmermann et al. 2018). Additionally, AZM at subinhibitory concentrations can suppress the expression of quorum-sensing–regulated virulence factors (Nalca et al. 2006). While some studies suggest that AZM may exhibit antimicrobial effects, its activity against stationary-phase *P. aeruginosa* cells is less well understood (Tateda et al. 1996). Recently, studies demonstrated the effects of specific mutations of ribosomal proteins uL4 (*rplD*, PA4262) and uL22 (*rplV*, PA4258) on the resistance of *P. aeruginosa* to macrolides by isolating the specific contribution of each mutation within a model strain background (Goltermann et al. 2022, 2024). These mutations were identified in clinical strains and enhanced the resistance of the model strain, prompting a re-evaluation of macrolide usage and its intended applications.

### Ribosomal RNA methylation

Ribosomal protection by methylases [rRNA Methyl Transferases (RMTases)] is a target‐alteration mechanism that prevents antibiotic binding. In *P. aeruginosa*, the most‐documented 16S site is N7‐G1405, modified by acquired RMTases such as RmtA-H family enzymes and ArmA. The modification of this position confers high‐level resistance to 4,6‐disubstituted 2‐deoxystreptamine aminoglycosides (amikacin, tobramycin, gentamicin, and kanamycin), but not to 4,5‐substituted 2‐deoxystreptamine aminoglycosides (streptomycin and spectinomycin). Structural studies have shown that the added methyl group at G1405 sterically clashes with the binding pocket of 4,6-aminoglycosides whereas 4,5‐DOS drugs approach the ribosome at a different angle (Wachino et al. 2020).

By contrast, methylation of N1‐A1408 by enzymes such as NpmA blocks both 4,6‐ and 4,5‐substituted aminoglycosides, as well as apramycin, although, to the best of our knowledge, A1408‐modifying RMTases have not been identified in *P. aeruginosa* (Wachino et al. 2020). Importantly, acquired 16S‐RMTases act on the mature 30S subunit, recognizing structural features that form only after ribosome assembly (Wachino et al. 2010). Despite this modification, ribosome function is largely unimpaired, and no major fitness cost is usually observed, which explains the persistence of these enzymes in clinical isolates (Wachino et al. 2020). A recent study showed that among 221 *P. aeruginosa* isolates highly resistant to amikacin, gentamicin, and tobramycin, 16S RMTase genes were detected in only 8.6% of isolates, indicating that 16S RMTases are not a predominant mechanism of aminoglycoside resistance in this species (Taylor et al. 2022).

Finally, the core methyltransferase KsgA, which normally dimethylates A1518/A1519 in 16S rRNA, modulates susceptibility in *P. aeruginosa* to kasugamycin and hygromycin B: loss of *ksgA* increases resistance to kasugamycin and sensitivity to hygromycin B, likely due to alterations in decoding‐site conformation (Phatinuwat et al. 2024).

Methylation of the 23S rRNA is uncommon in *P. aeruginosa* but may occur through horizontal acquisition of *erm* genes as has been demonstrated for *rmt*A genes (Yokoyama et al. 2003). Indeed, heterologous expression of *Clostridium perfringens ermBP* in *P. aeruginosa* methylates the A2058 position (*Escherichia coli* numbering), preventing macrolide binding in the peptide exit tunnel and abolishing the effects of AZM on virulence factor production (Köhler et al. 2007). Endogenous *erm* genes have not been widely reported, and macrolide resistance in *P. aeruginosa* more often arises from efflux or permeability barriers.

Taken together, these findings demonstrate that while the chemistry of methylation (G1405 vs. A1408 vs. A1518/A1519, or A2058 in 23S) defines the resistance spectrum, the unifying outcome is the maintenance of ribosomal function by preventing antibiotic engagement. Because acquired 16S RMTases confer pan‐resistance to nearly all clinically used aminoglycosides, including next‐generation agents such as plazomicin, they represent a major global threat that warrants global surveillance (Wachino et al. 2020).

### Ribosome protection proteins

RPP are a key component of the maintenance of the active ribosome pool. They act differently from the mutations and methylations described in the previous sections, as the ribosomes remain unmodified.

**Figure 2:**
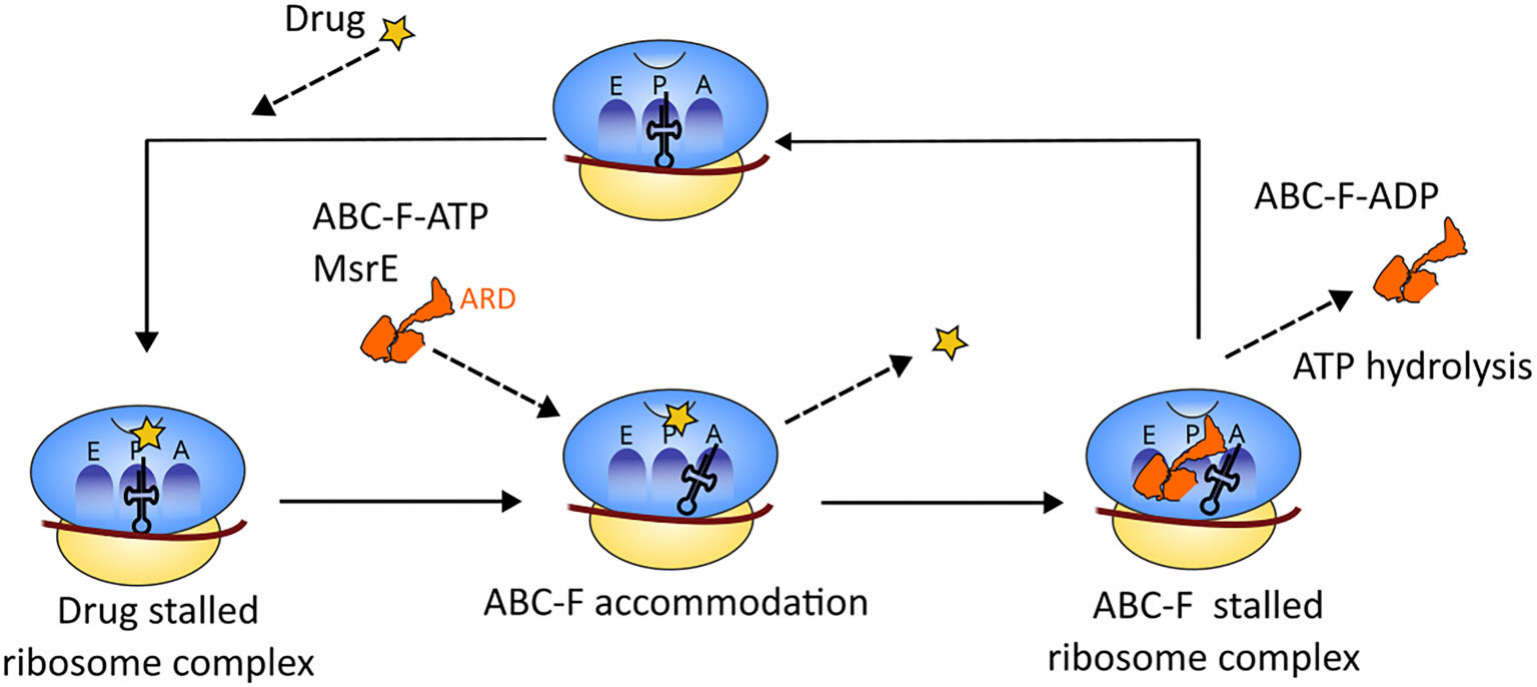
This figure illustrates the mechanism of an ABC-F protein displacing an antibiotic from the ribosome to allow translation to resume. The ARD (Antibiotic Resistance Domain) of the ABC-F extends into the antibiotic-binding site and triggers the release of the antibiotic. ATP hydrolysis then induces the dissociation of the ABC-F from the ribosomal complex.

ABC-F proteins belong to a superfamily of ATPases widely distributed among eukaryotes and bacteria. Members of this family function as translation factors, interacting with the ribosome exit site (E-site) to alter the conformation of its peptidyl-transferase center (PTC). These proteins support canonical protein synthesis under various stress conditions. A specific subgroup of bacterial ABC-F proteins, known as Antibiotic Resistance (ARE ABC-F) proteins, promotes resistance to antibiotics that target the 50S ribosomal subunit, including lincosamides, pleuromutilins, and macrolides (Ero et al. 2021, Fostier et al. 2021). In *P. aeruginosa*, this mechanism is essentially carried out by the ABC-F protein "MsrE". MsrE not only abolishes the effect of azithromycin *in vivo* (Ding et al. 2018), it can also confer macrolide resistance in *E. coli* cells as well as the cell-free translation assay (Su et al. 2018). MsrE provides *P. aeruginosa* with resistance to the inhibitory effect of AZM on quorum sensing, by dislodging this antibiotic from the ribosome (Ding et al. 2018, Su et al. 2018). The cryo-EM structure of *P. aeruginosa* MsrE bound to ribosome sheds light on its protection mechanism by drug displacement (Su et al. 2018). Briefly, the protein binds to the ribosomal exit site (E-site), whereby drug resistance is mediated by the antibiotic resistance domain (ARD) connecting the two ATP-binding domains. The ARD of E-site-bound MsrE extends toward the drug-binding region within the PTC and leads to conformational changes in the P-site tRNA acceptor stem, the PTC, and the drug-binding site, causing the release of corresponding drugs (Ero et al. 2021) (Fig. 2).

Four other ABC-F are encoded in the core genome of *P. aeruginosa*, namely PA1964, PA4595, PA3019, and PA1425, but their function remains to be determined. They share sequence similarities with known ABC-F proteins from *E. coli* (YbiT, EttA, Uup, and YheS, respectively) associated with a specific rescue process such as translational arrests due to polyproline, poly-basic, or poly-acidic sequences (Chadani et al. 2024). Among them, the ABC-F protein encoded by PA1964 in *P. aeruginosa* PAO1 may be worth investigating in the context of ribosome protection, as it shows high homology with the ARE-ABC-F VgaL (Lmo0919) from *Listeria monocytogenes*. VgaL works in coordination with HflxR, a variant of HflX, to induce drug displacement of lincomycin or erythromycin from the ribosome (Koller et al. 2022). *P. aeruginosa* PAO1 also possesses an HflX homolog, encoded by PA4943, whose function remains unexplored. HflX is a GTPase associated with ribosome recycling, 50S subunit biogenesis, and ribosome hibernation in *E. coli* (Zegarra et al. 2023). In *P. aeruginosa*, the expression of this gene appeared to be upregulated by a cellular increase in the sigma factor RpoH and by heat shock, reinforcing its role in cell homeostasis upon stress (Williamson et al. 2023). PA4943 was also identified by transposon sequencing as a gene important for resistance to colistin (Vitale et al. 2020).

## Ribosome rescue

**Figure 3:**
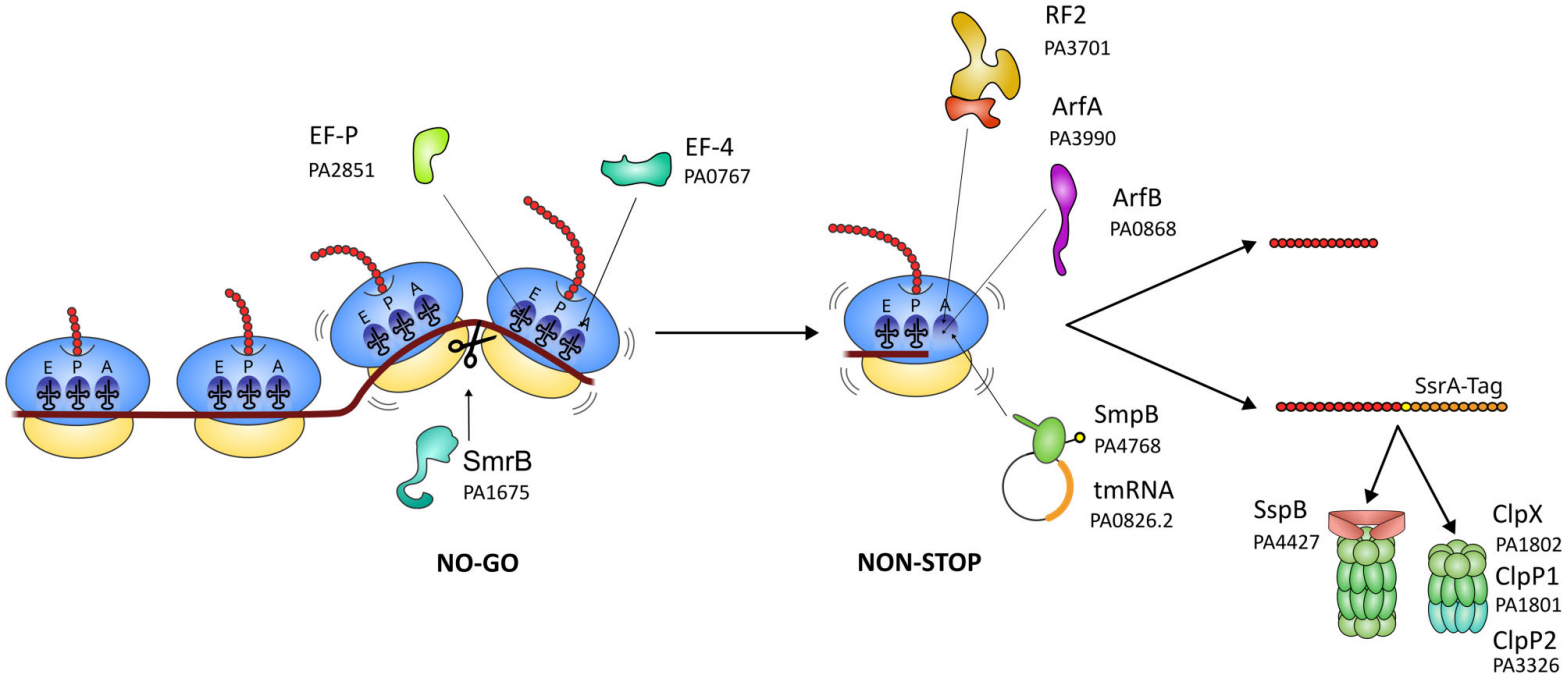
Schematic summary of the key players involved in ribosome rescue. The gene codes provided correspond to *P. aeruginosa* PAO1. It is worth noting that SsrA-tagged substrates from tmRNA-SmpB rescue process are primarily directed to the ClpXP/SspB complex for degradation, whereas the peptides released by the ArfA or ArfB systems are not targeted for degradation. See text for details. E: Exit site; P: Peptidyl-tRNA binding site; A: Aminoacyl-tRNA binding site.

During canonical translation, ribosome stalling can occur due to mRNA structures, amino acid sequence constraints, tRNA shortages, mRNA truncation, translation inhibitors, or cellular stress, all of which disrupt ribosomal progression and require resolution mechanisms (Buskirk and Green 2017). These situations, if not resolved rapidly, are detrimental to bacterial survival because they sequester part of the ribosome pool and lead to the synthesis of incomplete, potentially toxic proteins. Fig. 3 provides an overview of the ribosome rescue systems identified in *P. aeruginosa*.

### Resuming translation of no-go translational complexes

When ribosomes stop or significantly slow down during translation, before the stop codon is reached, the resulting complex is referred to as a no-go complex. Multiple factors can promote translation attenuation to the extent of ribosome stalling, including certain leader peptides and peptide sequences, specific codon sequences, or antibiotics (De Valdivia and Isaksson 2005, Gong et al. 2007, Jacinto-Loeza et al. 2008). To mitigate the adverse effects of these situations, specialized translational accessory factors have evolved to resolve unwanted pauses to resume canonical translation, notably elongation factors .

When prolonged ribosome stalling occurs, other mechanisms act by converting the no-go complex into a non-stop complex (ribosome stalled at the end of a non-stop mRNA), thereby facilitating ribosome rescue pathways such as *trans*-translation.

The severity of no-go events is very variable, going from brief pauses inside Open Reading Frame that are rapidly resolved and mainly involve elongation factors, to no-go events where the ribosome is stalled, forming non-stop complexes. The latter need rescue pathways to be resolved.

It is interesting to note that two approaches exist in bacteria for rescuing stalled and collided ribosomes: either a disassembly of the leading 70S ribosome to allow downstream ribosomes to continue translation (RQC systems: primarily in Firmicutes) (Filbeck et al. 2022), or a cleavage of the mRNA upstream of the stalled ribosome followed by rescue (SmrB, trans-translation, or alternative systems: primarily in proteobacteria). Since the RQC system (i.e. RqcH and RqcP coding genes) is absent in *P. aeruginosa*, this system will consequently not be addressed in the present review.

#### Elongation factors

Polyproline stretches in nascent peptides cause translational pauses due to the rigid cyclic structure of the amino acid, which restricts backbone flexibility and introduces a sharp turn in the peptide chain. Elongation factor-P (EF-P) alleviates this issue by stabilizing peptidyl-tRNA in a productive conformation (Volkwein et al. 2019). Deletion of EF-P (PA2851) is not lethal in *P. aeruginosa* but creates a growth defect (Balibar et al. 2013) and an upregulation of *mexXY* efflux pump, an aminoglycoside-inducible multidrug transporter contributing to aminoglycoside resistance (Sobel et al. 2003). In general, EF-P requires a post-translational modification on a conserved residue. Even if the position of the modification is well conserved, the modification can differ from one organism to another.

The structural analyses of *P. aeruginosa* EF-P have shown that a post-transcriptional modification, the attachment of a single cyclic rhamnose moiety, occurs on an Arg residue. In *E. coli*, the modification occurring on the same residue is a β-lysylation and is catalyzed by PoxA and is essential for EF-P activity (Yanagisawa et al. 2010). Genome analysis of organisms that lack *poxA* but have a modified Arg in EF-P, like *P. aeruginosa*, revealed the presence of a highly conserved glycosyltransferase performing the rhamnosylation instead of lysylation, named EarP (PA2852). Furthermore, the gene coding for this protein is next to the gene coding for EF-P. Both *efP* and *earP* mutants of *P. aeruginosa* displayed a deficiency in swimming motility and an increased sensitivity to cell wall-targeting antibiotics, further establishing the tight link between these two proteins (Rajkovic et al. 2015).

Certain challenging conditions such as high magnesium concentrations, low pH, or low temperatures can also have a detrimental effect on canonical translation. The translation elongation factor, EF-4 (also named LepA), is thought to help mitigate these effects, but its exact role remains to be thoroughly investigated. EF-4 contains a GTPase domain and may function as a back-translocase by catalyzing the backwards movement of tRNAs from the POST state (tRNAs in P and E-sites) to the PRE state (tRNAs in A and P-sites). This action is in direct opposition to the function of EF-G (Qin et al. 2006, Heller et al. 2017). In *P. aeruginosa*, EF-4 is hypothetically encoded by PA0767 and further research is needed to evaluate its specific contribution to translational control.

#### Transition from no-go to non-stop caused by stress response

During bacterial amino acid starvation, ribosomes stall on intact mRNAs because uncharged tRNAs bind to their A-sites, triggering the stringent response. The stringent factor RelA binds to ribosomes and initiates (p)ppGpp synthesis. This process downregulates the transcription of proteins and RNAs involved in translation while upregulating the transcription of enzymes involved in amino acid synthesis (see Bange et al. 2021, for a review). Ten type II toxin–antitoxin (TA) systems have been found in *P. aeruginosa*, including ribosome-associated ribonucleases of the RelE superfamily (Li et al. 2023). Reduction of protein synthesis rates, combined with the ATP-dependent activity of Lon protease, diminishes the amount of RelB, part of the RelBE TA system (Christensen et al. 2001). These systems consist of a stable toxin (RelE) neutralized by a labile antitoxin (RelB). When RelB concentration decreases, the stable RelE is freed and binds to the stalled ribosome, specifically cleaving mRNA in the A-site (Christensen and Gerdes 2003). This creates a vacant A-site, converting these complexes into non-stop stalled ribosomes, thus becoming a substrate for ribosome rescue. The expression of *relBE* enhances persister cell formation in biofilms when exposed to ciprofloxacin and colistin in *P. aeruginosa* (Golmoradi Zadeh et al. 2022). It also influences biofilm formation by indirectly regulating the expression of genes associated with biofilms (Mahmoudi et al. 2022). However, the conserved annotated RelBE system in *P. aeruginosa* genomes was recently revealed as a ParDE-type TA system, involved in inhibition of gyrase-mediated supercoiling of DNA (Muthuramalingam et al. 2019). Another ribotoxin, HigB (PA4674.1), reduces the production of various virulence factors such as pyochelin and pyocyanin production, and interferes with swarming motility and biofilm formation (Wood and Wood 2016), but its role in ribosome rescue after a no-go event remains to be evaluated. The tmRNA system is essential for bacterial survival under stress conditions induced by mRNase toxins. By rescuing stalled ribosomes and degrading aberrant proteins, tmRNA may help maintain cellular homeostasis and reduce the cytotoxic effects of these toxins (Christensen et al. 2001, 2003).

It should be noted that other mechanisms may operate to promote the transition from a no-go to a non-stop complex. The gene PA1675 of strain PAO1 encodes a predicted SmrB-like protein containing conserved SMR (Small MutS related) domains, similar to *E. coli* SmrB, *Saccharomyces cerevisiae* Cue2, and *Bacillus subtilis* MutS2 (InterPro analysis). SmrB is the nuclease responsible for cleaving the mRNA between the stalled and collided ribosomes (Leedom and Keiler 2022, Saito et al. 2022). Exploring the function of this SmrB-like protein in *P. aeruginosa* would help clarify whether similar ribosome rescue pathways operate in this species.

Other splitting factors may be involved in resolving complexes formed by collided ribosomes. Among them, HrpA is an RNA helicase known to rescue stalled ribosomes in *E. coli* (Campbell et al. 2025). Interestingly, PA3297, annotated in the PAO1 genome as a probable ATP-dependent helicase HrpA, was reported to counteract the effects of the ribosome stalling antibiotic AZM in *P. aeruginosa* (Tan et al. 2016).

### Translation stalling—non-stop translational complexes

When ribosomes reach the end of a truncated mRNA without encountering a termination codon, or after no-go complexes are converted to non-stop (see above), they accumulate on the defective mRNA and form a stalled polysomal chain (Cougot et al. 2014), forming non-stop translational complexes.

#### *Trans*-translation mediated rescue

In *P. aeruginosa*, as in all bacteria, *trans*-translation is the main cellular process involved in the rescue of ribosomes stalled on non-stop mRNAs, forming non productive translation complexes. It adds a degradation tag to the C-terminus of the incomplete polypeptide and allows the canonical recycling of all the components. This system is composed of two partners that form a 1:1 complex: SmpB (PA4768) and the transfer-messenger RNA (tmRNA, encoded by the *ssrA* gene, PA0826.2). EF-Tu•GTP binds to the alanyl-tmRNA-SmpB complex to bring it to the vacant A-site of a stalled ribosome. Stalled ribosomes are selected by SmpB, which uses its C-terminal tail to probe the occupancy of the ribosomal mRNA channel inside and downstream from the decoding site. The incomplete peptide is then transferred onto the tRNA-like domain of the tmRNA, which is aminoacylated with an alanine. Translation then resumes on the mRNA-like domain of the tmRNA, thereby allowing canonical ribosome recycling because of the stop codon present at the end of this sequence. These main states of *trans*-translation have been described in *E. coli* using cryo-EM (Rae, Gordiyenko and Ramakrishnan 2019, Guyomar et al. 2021, D’Urso et al. 2023), giving a sequential insight of its organization. This has not been done in *P. aeruginosa* yet, and this work on another model organism could reveal functional specificities of *trans*-translation systems, particularly at the interface between SmpB and tmRNA or in the recognition of the mRNA channel by the C-terminal tail of SmpB.

In *P. aeruginosa*, the sequence of the tag is ANDDNYALAA and does not differ from other tags for the residues interacting with the proteases ClpXP or ClpAP (Flynn et al. 2001). The stringent starvation protein B (SspB, PA4427) binds specifically to the ANDDNY domain of the SsrA-tagged substrates and enhances the recognition of these proteins by the AAA + ATPase ClpX, while LAA region interacts with ClpX (Wah et al. 2002, McGinness et al. 2006). This tag is conserved among the available sequences of *P. aeruginosa* but some differences in length and sequences exist in the *Pseudomonas* genus. *Trans*-translation relies on the functionality of the peptidase ClpP_1_ (PA1801) for the nascent peptide to be degraded (Hall et al. 2017). *P. aeruginosa* has two homologues of ClpP, ClpP_1_ and ClpP_2_ (PA3326), which can form two types of complexes with ClpX: ClpXP_1_ and ClpXP_1_P_2_. The *clpP_1_* gene is expressed throughout growth, whereas *clpP_2_* is tightly controlled by quorum-sensing signaling molecules and the LasR transcription factor (Mawla et al. 2021).

*Trans-*translation plays an essential role in *P. aeruginosa* tolerance to AZM and multiple aminoglycoside antibiotics (Ren et al. 2019). Accordingly, suppression of *trans-*translation, either by deleting or mutating SmpB or tmRNA, has an impact on the persistence of *P. aeruginosa* treated by ribosome-targeting antibiotics (Morita et al. 2006, Ren et al. 2019). A whole-genome approach using a random library of 57 360 Tn*5* mutants in *P. aeruginosa*, screened *in vitro* for swarming, exoprotease and pyocyanin production, and toxicity in several models (*Drosophila melanogaster*, *Caenorhabditis elegans*, human cell lines, and mice), revealed that the *smpB* mutant of *P. aeruginosa* PAO1, unable to perform *trans-*translation, displayed reduced swarming motility, pyocyanin production, and virulence in *C. elegans* compared with wild-type (Dubern et al. 2015). Considering this information, *trans*-translation emerges as an appealing target for antimicrobial drug design against *P. aeruginosa*.

#### Alternative rescue factors mediated rescue

If *trans*-translation is overwhelmed or disrupted, alternative rescue factors ArfA and ArfB may take over in *P. aeruginosa* (Abo and Chadani 2014). This is why the deletion of *ssrA* or *smpB* is not lethal in this bacterium. ArfA functions by detecting stalled ribosomes on non-stop mRNAs. Its C-terminal tail inserts into the vacant mRNA channel downstream of the A-site, which allows it to stably bind only when no mRNA is present in this part of the channel. Once anchored, the N-terminal region of ArfA recruits Release Factor 2 (RF2). In the absence of a stop codon, RF2 is normally inactive, but binding to ArfA stabilizes RF2 in the active conformation, allowing its catalytic GGQ motif to hydrolyze the peptidyl-tRNA bond and release the stalled polypeptide (Kurita et al. 2020).

ArfA is thought to be regulated by *trans*-translation. Indeed, ArfA mRNA forms a hairpin near the end of the sequence, which is recognized and cleaved by RNase III to form a non-stop mRNA that is translated into the active form of ArfA, that is, if not tagged or degraded by *trans*-translation (Garza‐Sánchez et al. 2011). When it is not truncated, the protein is thought to be unstable in the cell because of the presence of a hydrophobic C-terminal tail (Chadani et al. 2011). In *P. aeruginosa*, ArfA (PA3990) features a hydrophobic C-terminus and a predicted upstream hairpin structure, suggesting a regulatory pattern like that of *E. coli*. However, the hairpin structure of PA3990 mRNA is shorter than the known substrates of RNase III, and there are no published data on the impact of this difference (Schaub et al. 2012). PA3990 expression has been shown to be upregulated by tobramycin when *P. aeruginosa* is present in a biofilm on airway epithelium cells, along with *arfB* and *prfH* (Anderson et al. 2008), suggesting a possible role in adaptation to antibiotics targeting the ribosome in this bacterium.

The other known alternative rescue factor is ArfB. The protein harbors the GGQ motif responsible for the peptidyl-tRNA hydrolase activity (Burroughs and Aravind 2019), but differs from RF1 and RF2 by the fact that it is unable to bind to the ribosome unless the mRNA channel of the ribosome is empty (see Müller et al. 2021, for a review). It has a combination of ArfA-RF2 functions. However, ArfB alone can only restore growth of the tmRNA/*arfA* double mutant of *E. coli* when it is overexpressed, suggesting that its function may not be limited to serving as a back-up for *trans*-translation (Chadani et al. 2011). *P. aeruginosa arfB* (PA0868) remains incompletely characterized and further research is needed.

## Hibernation: hibernation-promoting factor and uncharacterized proteins

**Figure 4:**
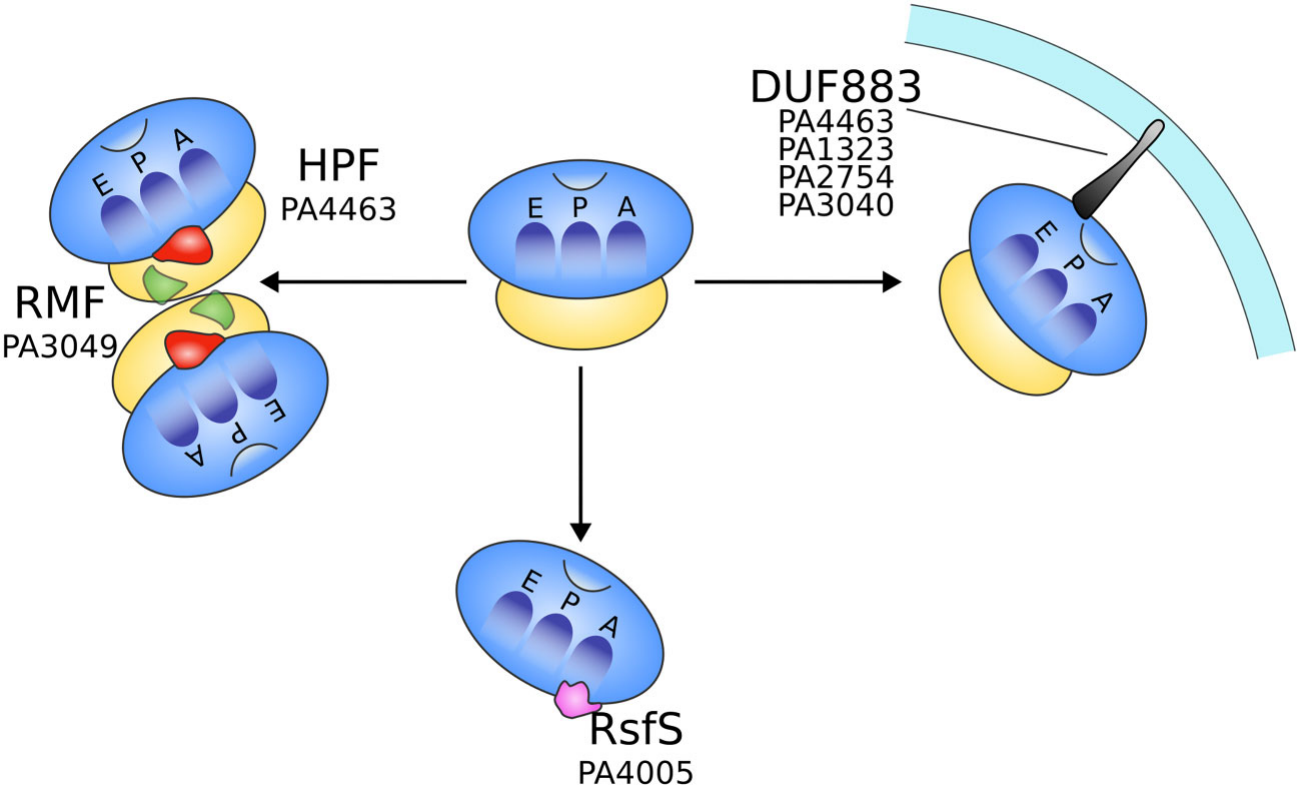
Schematic summary of the proteins involved in ribosome hibernation. The gene codes provided correspond to *P. aeruginosa* PAO1. HPF, RMF, RsfS, and YqjD-homologs (DUF883) are key factors in ribosome hibernation. RMF and HPF promote 70S ribosome dimerization into inactive 100S particles, reducing translational activity under stress. RsfS may bind the 50S subunit, preventing its association with the 30S subunit and further inhibiting translation. YqjD-like proteins may anchor ribosomes to the membrane and contribute to their stabilization in the hibernating state. See text for details. E: Exit site; P: Peptidyl-tRNA binding site; A: Aminoacyl-tRNA binding site.

Ribosome hibernation is a mechanism in which proteins bind to fully functional idle ribosomes to sequester them for later use in *P. aeruginosa*. These proteins can be present in the cytoplasm or can be integrated into the inner membrane. During periods of starvation and stress, ribosomes detach from mRNA and tRNAs, thus becoming substrates for ribosome hibernation factors. These factors inhibit protein synthesis by occupying the binding sites on ribosomes for mRNA and tRNAs, and protect ribosomes from degradation by shielding their vulnerable active centers from cleavage by cellular nucleases, while also preventing protein synthesis (Williamson et al. 2012). Fig. 4 summarizes the mechanisms of ribosome storage in inactive forms identified in *P. aeruginosa*, either experimentally or through *in silico* genome analyses.

### Hibernation inducing cytoplasmic proteins

Hibernating ribosomes are primarily formed by the assembly of 100S particles (see Prossliner et al. 2018, for a review). Upon nutrient starvation in gammaproteobacteria, the ribosome modulation factor (RMF) binds near the 30S head and blocks mRNA entry, inducing conformational changes that allow a second ribosome to dock through the 30S–30S interface. The hibernation-promoting factor (HPF) then stabilizes this interaction by inserting into the A- and P-sites, producing the translationally inactive 100S dimer (Trösch and Willmund 2019). RMF is a soluble protein able to bind to the ribosome, forming immature 90S. Then, with the help of HPF, the mature 100S is produced. HPF blocks ribosome degradation by masking the cleavage position on the 23S rRNA. The ribonuclease responsible for the degradation of the ribosome in the absence of HPF has not yet been characterized in *P. aeruginosa*, but it is RNase E in *E. coli* (Sulthana et al. 2016). Although in *E. coli* RMF functions as an initiator, in association with HPF, for a two-steps process to form a dimer of 100S via a 90S inactive intermediate, there is no evidence of a 90S intermediate in *P. aeruginosa*.

*P. aeruginosa* HPF-encoding gene (PA4463) is located downstream of *rpoN*. They form a putative operon, but PA4463 has its own promoter displaying a *rpoD*/*rpoS*-like structure. *hpf* transcripts were shown to be abundant in the dormant antibiotic-tolerant cells in biofilms (Franklin et al. 2020). Wild-type planktonic *P. aeruginosa* PAO1 cells survived for many days in nutrient-depleted medium without significant loss of viability while *hpf* mutant cells displayed reduced viability over time and reduced ribosome content. The starved Δ*hpf* strain also displayed heterogeneity in colony size and increased lag time for the subpopulation of cells that survived starvation. These phenotypes were not observed in the starved Δ*rmf* mutant. HPF, but not RMF (PA3049), is required for ribosome preservation and resuscitation of starved *P. aeruginosa* cells (Akiyama et al. 2017) by preventing the degradation of rRNA and ribosomal proteins L5 and S13 (Theng et al. 2020). *P. aeruginosa hpf* expression seems to be only moderately affected by the stringent response, RpoS or DksA2, and its 5’-UTR structure affects the expression due to the presence of a hairpin structure (Akiyama et al. 2018). While *P. aeruginosa* processes both RMF and HPF, it does not seem to have an *E. coli* YfiA-homolog, which can form inactive 70S structures. Rather, the C-terminal structure of *P. aeruginosa* HPF exhibits an intermediate length between *E. coli* YfiA and *E. coli* HPF (Akiyama et al. 2018, Prossliner et al. 2018, Franklin et al. 2020). Even if it is mentioned in Fig. 4 by prediction or by hypothesis with mechanisms evidenced in bacterial models, it must be noted that the role of RMF in *P. aeruginosa* remains elusive, just like the different intermediates in the inactivation of the ribosomes. New insights into the role of RMF (new proposed name BatR) seem to point towards a role in virulence modulation, in part via the Stress Response Kinase A (PA0486) (Piazza et al. 2025). The structures of ribosomes with HPF and/or RMF in their active sites remain to be solved for *P. aeruginosa*.

The ribosome silencing factor S (RsfS) binds to the L14 protein at the intersubunit interface of the 50S, sterically preventing the association of 30S and 50S subunits during the stationary phase (Prossliner et al. 2018). This gene has been identified bioinformatically (PA4005 in *P. aeruginosa* PAO1; PA14_12 030 in *P. aeruginosa* PA14), but no dedicated study has been published. Nevertheless, PA4005 has been shown to be overexpressed in the CF *P. aeruginosa* AES-1R epidemic strain (Scott et al. 2013) and to be important for swarming motility and optimal growth in *P. aeruginosa* PA14 (Yeung et al. 2009).

### Hibernation inducing membrane proteins

The DUF883 family comprises proteins involved in the sequestration of ribosomes into the membrane. The three known members with this function in *E. coli* are ElaB, YqjD, and YgaM, with putative *P. aeruginosa* homologues being encoded by PA1323, PA3040, and PA2754, as determined by protein sequence comparison on pseudomonas.com (Table 1). PA1323 and PA3040 expression increase under membrane stress (Sanz-García et al. 2019) and are part of the σ^22^ regulon, also involved in the membrane stress response (Wood and Ohman 2012). PA3040 has been shown to be expressed 100 times more in MDR *P. aeruginosa* (Montemari et al. 2022). To date, little is known about the function of PA2754 product. Of these three proteins, none has been the subject of structural studies identifying their ribosome-binding ability. Preliminary studies have identified YqjD, a protein expressed during the stationary phase that contains a transmembrane motif in its C-terminal region and a ribosome interaction domain with 70S and 100S ribosomes in its N-terminal region. Overexpression of *yqjD* in *E. coli* leads to inhibited cell growth, suggesting that during the stationary phase YqjD may direct ribosomes to the membrane (Yoshida et al. 2012). This conserved hypothetical protein is also encoded by the *P. aeruginosa* genome (in PAO1: PA3040). In this species, however, PA3040 is proposed to play a role in maintaining cell envelope homeostasis in sessile cells (Wood and Ohman 2012). Recently, PA3040 was found to contribute to tobramycin resistance in clinical isolates of *P. aeruginosa* but not in the model strain PAO1 (Østergaard et al. 2024). The sequestration of inactive ribosomes at the membrane may be a conserved preservation mechanism during slow growth and could serve as a target to reduce pathogen fitness, but further investigations are needed to identify such a mechanism in *P. aeruginosa*.

**Table 1:** Genes and proteins associated with translation fail-safe and ribosome preservation mechanisms in *Pseudomonas aeruginosa* PAO1. Entries include experimentally characterized factors and predicted homologues identified by sequence similarity to known proteins in other bacteria. Genes not discussed in the text because of a lack of information specific to *P. aeruginosa*, but potentially of interest, are marked with an asterisk (*).

Product name	Gene code
ABC-F (close to EttA)	PA4595
ABC-F (close to Uup)	PA3019
ABC-F (close to YbiT)	PA1964
ABC-F (close to YheS)	PA1425
MsrE	-
Alternative rescue factor A (ArfA)	PA3990
Alternative rescue factor B (ArfB)	PA0868
ClpP1	PA1801
ClpP2	PA3326
ClpX	PA1802
Elongation Factor P (EF-P)	PA2851
Elongation Factor 4 (EF-4; also named LepA)	PA0767
HflX	PA4943
Hibernation Promoting Factor (HPF)	PA4463
Hibernation promoting protein (DUF883 family)	PA1323
Hibernation promoting protein (DUF883 family)	PA2754
Hibernation promoting protein (DUF883 family)	PA3040
HrpA ATP dependent Helicase	PA3297
*PrfA (ArfB_2)^1^	PA4665
PrfH1	PA5470
Release Factor 2 (RF2)	PA3701
Ribosomal protein uL6	PA4248
Ribosome Modulating Factor (RMF)^2^	PA3049
Ribotoxin HigB	PA4674.1
RsfS	PA4005
SmpB	PA4768
SmrB or SmrA	PA1615
tmRNA	PA0826.2

Notes: ^1^Alternative ribosome rescue factor releasing peptidyl-tRNA from ribosomes stalled on truncated mRNAs; ^2^ Putative function.

## Concluding remarks

This review aimed to enhance the understanding of translation quality control mechanisms in the medically relevant pathogen *P. aeruginosa*. These mechanisms, which maintain the fitness and virulence of the bacterium, represent promising targets for the development of new antimicrobial strategies. In addition to summarizing recent findings on *P. aeruginosa*, this review highlighted potential research avenues inspired by studies on other bacterial species, integrated with insights from the genomic analysis of *P. aeruginosa*. It emphasizes areas where further research is needed to elucidate the complex processes employed by *P. aeruginosa* to maintain the quality and functionality of its translation machinery under environmental stress. More specifically, it identifies key factors involved in the protection, rescue, and preservation of ribosomes, as well as their roles in resistance to ribosome-targeting antibiotics and in virulence. Gaining a deeper understanding of the ribosome rescue mechanisms in *P. aeruginosa* and related bacteria may provide valuable insights into the unique patterns of antibiotic resistance in those bacteria, ultimately aiding in the development of more effective antibiotic treatments targeting translational quality control.
